# Synergistic Performance and Reaction Mechanisms of a Carbide Lime-Powdered Glass Composite for Soil Stabilization

**DOI:** 10.3390/ma19050837

**Published:** 2026-02-24

**Authors:** Yao Zhang, Zijie Feng, Yangfei Wu, Degang Liao, Xinyu Fan, Yu Xi

**Affiliations:** 1 College of Civil Engineering, Xijing University, Xi’an 710123, China; xjfzj2026@163.com (Z.F.); xjwyf2026@163.com (Y.W.); ldggang@163.com (D.L.); xiyu@xijing.edu.cn (Y.X.); 2 Key Laboratory of Geotechnical Mechanics and Engineering, Ministry of Water Resources, Yangtze River Scientific Research Institute, Wuhan 430010, China; 3 PowerChina Northwest Engineering Corporation Limited, Xi’an 710065, China; fanxy@nwh.cn

**Keywords:** soil stabilization, dispersive soil, cementitious products, carbide lime (CL), powdered glass (PG), industrial by-products

## Abstract

Carbide lime (CL) and powdered glass (PG), as industrial by-products, possess significant potential as eco-friendly soil amendment materials. This paper presents a systematic investigation into the effectiveness and reaction mechanisms of a composite material comprising CL and PG for stabilizing dispersive soils. A systematic experimental program was designed with varying CL (0.5–6.5%) and PG (4–16%) contents, along with curing ages of 1, 7 and 14 days. Macroscopic properties, including dispersibility and permeability, were evaluated through pinhole, mud ball, and permeability tests, while phase composition and microstructural evolution were analyzed using X-ray diffraction (XRD) and scanning electron microscopy (SEM). Results demonstrate a pronounced synergistic effect between CL and PG at optimal ratios: soil dispersibility is markedly improved when CL ≥ 2.5% and PG ≥ 8%, non-dispersive behavior is achieved at all curing ages with CL between 4.5 and 6.5% and PG between 4 and 16 permeability coefficient decreases significantly with increasing material content; for instance, increasing CL from 2.5% to 6.5% (at 16% PG) reduces the permeability coefficient by over 50%. Microstructural analysis reveals that CL supplies Ca^2+^ and an alkaline environment, whereas PG provides reactive SiO_2_ and Al_2_O_3_. Their interaction facilitates ion exchange and pozzolanic reactions, leading to the formation of C–S–H and C–A–S–H gels. These cementitious products effectively fill pores and bond soil particles, thereby enhancing structural stability. This study confirms that the CL-PG composite is an efficient and sustainable soil stabilization material. It provides novel insights into the synergistic mechanisms and optimal dosage range, offering valuable theoretical and practical guidance for the resource utilization of industrial by-products in geotechnical engineering.

## 1. Introduction

Carbide lime (CL) and powdered glass (PG), as two representative industrial by-products, possess distinct yet complementary chemical characteristics that make them promising candidates for designing sustainable soil stabilization materials [1]. CL, a residue from acetylene production, is primarily composed of calcium hydroxide (Ca(OH)_2_), providing a readily available source of calcium ions (Ca^2+^) and a persistent alkaline environment. Powdered glass, derived from crushed waste glass, is rich in amorphous silica (SiO_2_) and exhibits significant pozzolanic activity under alkaline conditions [2]. Their synergistic interaction is expected to promote more extensive pozzolanic reactions, generating a greater quantity of cementitious phases such as calcium silicate hydrate (C-S-H) and calcium aluminosilicate hydrate (C-A-S-H). This process could more effectively cement soil particles, optimize pore structure and simultaneously improve mechanical performance and durability [3,4]. This waste-based composite strategy aligns with the principle of sustainable development by utilizing waste materials for soil treatment, thereby offering considerable environmental and economic benefits [5].

**Figure 1:**
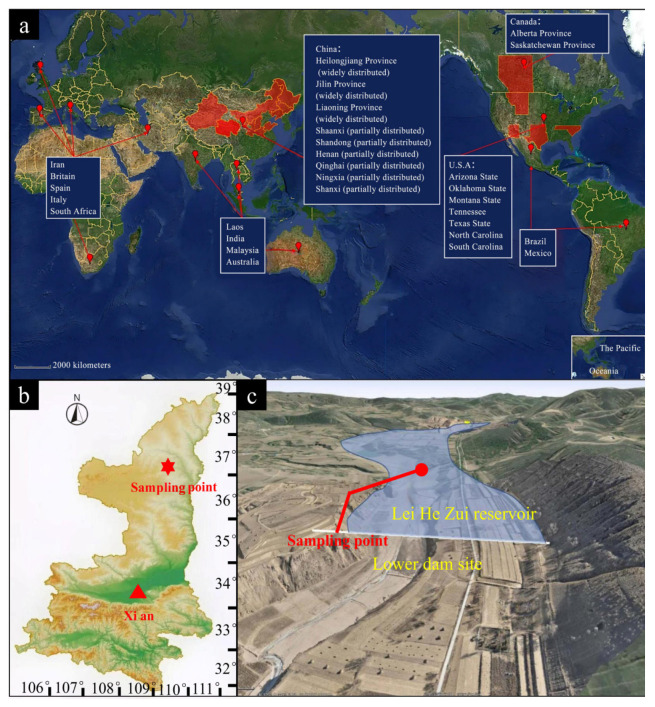
Test soil sampling site: (**a**) world distribution map of dispersive soils; (**b**) soil sampling locations for experiments in Shaanxi Province; (**c**) Lei He Zui Reservoir lower dam site.

Dispersive soils, characterized by the spontaneous deflocculation and transportation of their particles in static water, are a key factor triggering piping, seepage failure and internal erosion in dams, canals and road embankments, posing a severe threat to the long-term safety of infrastructure [6,7]. In China, the distribution of dispersive soils is primarily concentrated in 10 provinces in the northern region, including Qinghai, Xinjiang, Shaanxi and Inner Mongolia (Figure 1a). Conventional engineering treatments primarily rely on incorporating materials such as cement, quicklime, or fly ash into the soil to suppress its dispersiveness by enhancing strength and reducing permeability [8,9]. However, cement production is associated with high energy consumption and carbon emissions, while the sole use of lime may lead to excessive desiccation shrinkage and potential long-term strength retrogression [10]. Consequently, exploring the development of novel, green and efficient soil stabilization composite materials utilizing industrial solid wastes has become a significant frontier in geotechnical engineering and sustainable construction materials.

Current research on the use of single CL or PG for soil improvement has progressed. For instance, studies have confirmed the effectiveness of CL in stabilizing expansive and soft clays, with mechanisms similar to lime but offering greater economic advantages [6,10]. Concurrently, research on PG as a supplementary cementitious material in concrete or a precursor for geopolymers is extensive, validating its pozzolanic activity [11]. However, existing studies predominantly focus on single materials or composites of CL/PG with other systems (e.g., fly ash, slag). Systematic research specifically dedicated to the combination of these two wastes (CL and PG) for stabilizing highly dispersive soils remains scarce. The quantitative influence of their optimal mix ratio and synergistic effects on key engineering properties of soil (such as dispersibility and permeability), as well as the correlative mechanisms between the resulting microstructural evolution and macroscopic performance, require further clarification [12,13].

Based on this, the present study aims to systematically investigate the improvement efficacy and reinforcement mechanisms of a CL-PG composite system on dispersive soil from the perspective of waste-based composite material design. The research involves designing composite material formulations with varying CL (0.5–6.5%) and PG (4–16%) contents, combined with different curing ages (1, 7, 14 days), to comprehensively evaluate changes in the dispersivity classification and permeability coefficient of the treated soil. Advanced techniques including X-ray diffraction (XRD), scanning electron microscopy (SEM) and quantitative pore image analysis are employed to elucidate the ion exchange and pozzolanic reaction processes induced by the CL-PG system, the characteristics of cementitious product formation and the patterns of microstructural evolution. Therefore, this study aims to: (1) systematically evaluate the synergistic stabilization effect of CL-PG composites on dispersive soils; (2) identify the optimal dosage range of CL and PG for dispersibility control and permeability reduction; (3) elucidate the underlying reaction mechanisms through microstructural and phase composition analysis; (4) provide a sustainable and efficient soil stabilization strategy utilizing industrial by-products.

## 2. Materials and Methods

### 2.1. Test Materials

**Figure 2:**
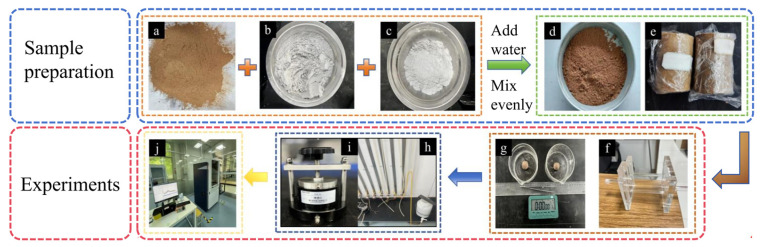
Diagrams of various experimental steps: (**a**) dispersive soil; (**b**) CL; (**c**) PG; (**d**) image of the improved soil mixture with dispersive soil; (**e**) soil sample wrapped in cling film; (**f**) pinhole test apparatus; (**g**) mud ball test apparatus; (**h**) complete permeability test setup with connected water tank; (**i**) TST-55 Permeability Tester (Nanjing Soil Instrument Factory Co., Ltd., Nanjing China); and (**j**) D8 ADVANCE X-ray Diffractometer (Bruker AXS GmbH, Karlsruhe Germany).

As illustrated in Figure 1b,c, the experimental soil samples were collected from the vicinity of the lower dam site of the Lei He Zui Reservoir in Jingbian County, Yulin City, Shaanxi Province, China. After being brought back to the laboratory, the soil samples were naturally air-dried, crushed and ground with a small wooden mallet and sieved through a 2 mm mesh for testing, as illustrated in Figure 2a.

The sieving process followed the standard dry sieving method, and the particle size distribution is presented in Table 1. The selected curing periods of 1, 7, and 14 days were chosen to capture the early-age reaction kinetics and the development of mechanical and hydraulic properties, which are critical for understanding the initial stabilization efficiency. Longer-term curing (e.g., 28 days) will be considered in future durability studies.

The physical properties of the samples were determined through standard tests, including compaction, liquid and plastic limit tests, particle size distribution analysis and relative density tests. The results of these measurements are presented in Table 1 and the mineral compositions of the soils are listed in Table 2.

In this experiment, CL was selected as one of the components of the calcium-based composite agent (Figure 2b). CL is an industrial by-product remaining from the acetylene production process. It is a light gray powdery substance accompanied by a pungent odor. Its primary mineral phase is Ca(OH)_2_, which contains active components similar to those of lime and readily reacts with water and volcanic ash materials in the soil. The raw material used in this experiment was obtained from Wuhu Environmental Technology Co., Ltd., in Zhengzhou city, China.

The ground PG (Figure 2c) serves as a source of amorphous silica, a substance whose reactivity in alkaline environments, such as Ca(OH)_2_ and NaOH, facilitates the occurrence of pozzolanic reactions. Such reactions lead to the formation of a gel-like phase, resulting in the generation of stable products that bind soil particles [14]. The PG used in this experiment was purchased from Xinlei Mineral Co., Ltd., in Shijiazhuang city, Hebei Province, China. The purchased PG was sieved using a 75-μm mesh to better activate the potential pozzolanic reactions.

To ensure uniform density throughout the height of the sample, carbide lime and glass powder were thoroughly mixed with the soil in specified proportions during the sample preparation process. Afterwards, the required amount of water was added proportionally, and the mixture was manually blended until homogeneous. Upon completion, the prepared samples were labeled, tightly wrapped in plastic film, and stored in sealed bags for further use.

### 2.2. Specimen Preparation

The soil samples that were screened through a sieve (Shaoxing Tianyun Instrument Equipment Co., Ltd., Shaoxing, China) were mixed with the stabilizing agents, with CL dosages of 0.5%, 2.5%, 4.5% and 6.5% and PG dosages of 4%, 8%, 12% and 16%. The mixed samples are denoted as CxPy (note: the sample notation “CxPy” uses “P” to denote “powdered glass,” not phosphorus.), where x and y represent the content of CL and PG, respectively. The curing periods were 1 day, 7 days and 14 days, which also corresponded to curing ages. The prepared specimens are illustrated in Figure 2d. All samples were prepared according to ASTM D4647-93 (2013) [15] standard to achieve a moisture content close to the optimum (±2%), with the amount of water added calculated using Equation (1).

(1) $$m_{w}=\frac{m}{1+0.01\omega_{0}}\times 0.01(\omega -\omega_{0})$$

where $m_{w}$ represents the mass of water added, m denotes the mass of the sample, $\omega_{0}$ indicates the initial moisture content of the sample and ω refers to the required moisture content for the test.

After the addition of water, the soil samples were sealed with plastic film and allowed to stand in containers for 24 h (Figure 2e). Subsequently, the corresponding mass of samples was weighed according to the standard dry density and compacted in five layers, with each layer subjected to roughening between compaction steps. The samples were compacted using a rammer to achieve a dry density greater than 95% of the maximum dry density, with the total height of the compacted samples reaching 38.1 mm [16].

The samples used in the mud ball test were prepared after the completion of the pinhole test, with the moisture content maintained near the liquid limit (±2%). Mud balls with an approximate diameter of 1.5 cm were produced. Both the pinhole and mud ball tests were performed to investigate the modification effect of calcium-based composites on dispersive soils [17]. The results of dispersibility determination from the two experiments may differ. Therefore, this study adopts the result with the highest dispersibility from both the pinhole and mud ball tests as the comprehensive criterion for judgment. The experimental procedure is detailed in Table 3.

### 2.3. Methods

Figure 2f illustrates the setup of the pinhole test apparatus. During the experiment, a small cone is inserted at the center of the water entry end of the sample. A 1 mm-diameter steel wire is passed through the soil sample and rotated to ensure that the pinhole remains unobstructed. Subsequently, the surface of the water entry end is sealed with wax to prevent the wax oil from blocking the pinhole hole. After assembling the experimental apparatus, the water entry end is connected to a rubber tube and flow tests are conducted under water heads of 50, 180, 380 and 1020 mm. The water flow rate per minute, the color change in the graduated cylinder and the morphological changes in the pinhole are recorded during the process.

Figure 2g illustrates the setup of the mud ball test. In this test, air-dried soil samples are sieved through a 2mm mesh and then mixed with deionized water to achieve a moisture content close to the liquid limit. Using tweezers, small balls are carefully placed at the center of a beaker containing 200 mL of deionized water. The dispersibility of the mud balls and the turbidity of the water are closely observed and recorded during the experiment. At time intervals of 5 min, 10 min, 30 min, 1 h, 3 h and 6 h, the disintegration of the mud balls is regularly documented to analyze their dispersibility behavior.

As illustrated in Figure 2i,h, the permeability test is conducted using a TST-55 Permeability Tester. During the testing process, the initial water head, height variations and time intervals are recorded and the water temperature at the drainage outlet is monitored. Samples are prepared according to the designed dry density, with water adjusted to the specified moisture content. The samples are then layered into a ring knife and cured for 14 days. During the evacuation saturation phase, the specimen is placed in a vacuum saturation chamber, where it is evacuated for 1.5–2 h. Subsequently, water is added to submerge the sample and evacuation is continued for an additional 2–2.5 h. The experiment is repeated at least three times and the permeability coefficient is calculated based on Equation (2), with the average of the measured data taken as the final value.

(2) $$k=2.3\frac{aL}{At}\mathrm{lg}\frac{H_{b1}}{H_{b2}}$$

where k represents the permeability coefficient of the specimen at a water temperature of 20 °C, 2.3 represents the conversion factor between the logarithms ln and lg, a denotes the inner cross-sectional area of the pressure pipe, L represents the height of the specimen, A refers to the surface area of the ring knife sample, t is the time interval between the two water head heights, $H_{b1}$ represents the initial water head height and $H_{b2}$ represents the water head height at time t.

For the X-ray diffraction (XRD) experiment, the typical samples (Table 3) were sent to the Materials and Energy Science and Technology Research Institute of Xijing University, where mineralogical composition analysis was performed using a D8 ADVANCE X-ray diffractometer (Figure 2j). X-ray diffraction (XRD) analysis was primarily employed for the qualitative assessment of the chemical composition and crystal structure of crystalline substances within solid powder samples. X-ray diffraction (XRD) analysis was performed on selected samples using a D8 ADVANCE X-ray diffractometer (Bruker AXS GmbH, Karlsruhe Germany) with Cu Kα radiation (λ = 1.5406 Å). The scanning range was set from 5° to 80° (2θ) with a step size of 0.02° and a scanning speed of 2°/min. The diffraction patterns were analyzed using MDI Jade 6.0 software, the PDF International Powder Diffraction Database [18], and the Chinese petroleum industry standard Method for Analysis of Clay Minerals and Common Non-Clay Minerals in Sedimentary Rocks by X-ray Diffraction (SY/T 5163-2018) [19] to identify the major mineral components of the specimens.

In this paper, the mean value method was applied for dimensionless normalization, as shown in Equation (3), Equations (4) and (5) were used to identify the maximum and minimum difference sequences. Finally, the correlation coefficients were calculated using the correlation coefficient formula, Equation (6) and substituted into Equation (7) to obtain the final gray relational degrees.

(3) $$\tilde{X_{i}}(k)=\frac{X_{i}(k)}{\overset{—}{X_{i}}},\;\overset{—}{X_{i}}=\frac{1}{n}\sum_{k=1}^{n}X_{i}(k),\;k\;=\;1,\;2,\;\ldots \;n$$

(4) $$\Delta_{i}(k)=\left|\tilde{X_{i}}\right.(k)—\left.\tilde{X_{1}}(k)\right|$$

(5) $$\Delta \;\min=\underset{i}{\min}\;\underset{k}{\min}\{\Delta_{i}(k)\},\;\Delta \;\max=\underset{i}{\max}\;\underset{k}{\max}\{\Delta_{i}(k)\}$$

(6) $$\xi_{i}(k)=\frac{\Delta \min+\rho \Delta \max}{\Delta_{i}(k)+\rho \Delta \max},\rho \;\in \;[0,\;1]$$

(7) $$\gamma_{i}=\frac{1}{m}\sum_{k=1}^{n}\xi_{i}(k)$$

Here, *ρ* is the distinguishing coefficient, set to 0.5. When *ρ* = 0.5, an association exists between the subsequence and the parent sequence if $\gamma_{i}$ ≥ 0.6; the association is considered weak if $\gamma_{i}$ < 0.6 and no meaningful association exists if $\gamma_{i}$ < 0.5. The synthesized compositions, namely C–A–S–H, C–S–H + C and C–S–H, are considered as comparative sequences $X_{i}$ = {$X_{i}(k)$, k = 1, 2, …, m; i = 1, 2, …, *n*}. Accordingly, a total of four comparative sequences (representing various factors) were chosen, with three experimental soil groups included in the analysis, yielding a total of 12 datasets. Thus, n = 3 and m = 4.

## 3. Results and Discussion

### 3.1. Macroscopic Mechanical Tests and Analysis

#### 3.1.1. Identification Test

**Figure 3:**
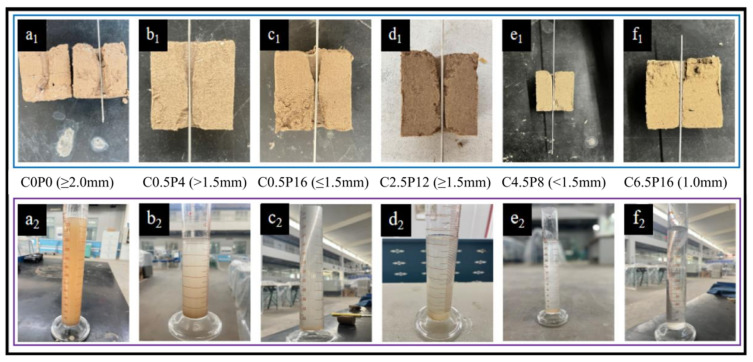
Pinhole test results. Row 1 shows the water inflow end of the specimen, and Row 2 shows the effluent after testing. Sample designations: (**a_1_**,**a_2_**) C0P0; (**b_1_**,**b_2_**) C0.5P4; (**c_1_**,**c_2_**) C0.5P16; (**d_1_**,**d_2_**) C2.5P12; (**e_1_**,**e_2_**) C4.5P8; (**f_1_**,**f_2_**) C6.5P16. **Note:** The first row shows the cross-sectional view of the sample specimen taken out from the pinhole test device and cut open. Row 2 shows the effluent after testing.

The results of the pinhole test are presented in Figure 3, clearly revealing the influence of CL and PG contents on soil dispersibility. The untreated pure soil sample (C0P0) exhibited typical characteristics of dispersive soil, with turbid effluent and significantly enlarged pores after testing (Figure 3a_1_). At low incorporation levels (e.g., CL 0.5% with PG 4–16%), the improvement effect was limited, and the samples were still classified as dispersive soil, showing turbid effluent and noticeable pore expansion (Figure 3b,c). When the CL content was increased to 2.5% combined with 12% PG, the soil behavior transitioned to that of transitional soil, with only slight turbidity observed in the effluent (Figure 3d). However, the most significant improvement was achieved when the CL content reached 4.5–6.5% with PG content within the range of 4–16%. Under this optimal mix proportion, all treated samples exhibited non-dispersive soil behavior. The key identifying features were that the effluent remained clear even under a high water head of 1020 mm, and no visible enlargement of the pinhole diameter was observed (Figure 3e,f). These results collectively demonstrate that the incorporation of the CL-PG composite effectively inhibits the dispersion of soil particles, and there exists a distinct threshold dosage (CL ≥ 4.5%, PG ≥ 4%) to achieve a stable non-dispersive state.

**Figure 4:**
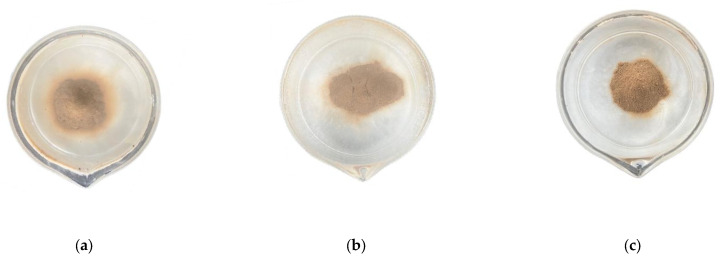

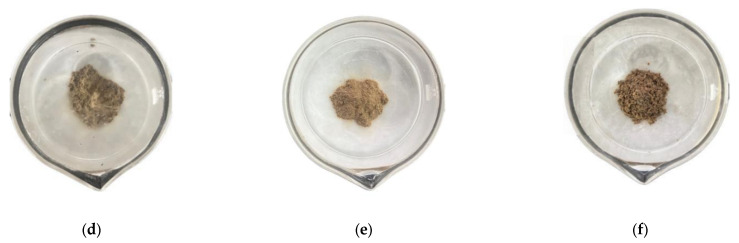
Mud ball test photo at 1 day of curing age: (**a**) C0P0; (**b**) C0.5P16; (**c**) C2.5P4; (**d**) C2.5P12; (**e**) C4.5P8; (**f**) C6.5P16.

As illustrated in Figure 4, the results of the ball clay test are as follows: When there was no amount of CL and PG, the mud ball rapidly disintegrated, the soil–water boundary was unclear and a thick “cloudy” suspension appeared at the bottom of the cup, classifying the sample as dispersive soil. When the amounts of CL and PG were 0.5% and 4%~16%, respectively (Figure 4b), the soil–water boundary was still unclear and a distinct cloudy colloidal suspension appeared on the surface or around the mud ball, classifying the sample as dispersive soil. When the amounts of CL and PG were 2.5% and 4%, respectively (Figure 4c), the soil–water boundary was only slightly unclear and slight turbidity from colloidal suspension appeared on the surface of the mud ball, classifying the sample as transitional soil. When the amounts of CL and PG were 2.5–6.5% and 8–16%, respectively (Figure 4b–f) and the soil–water boundary remained clear and no turbidity was observed upon contact between the mud ball and water, classifying the sample as nondispersive soil.

The results of the two tests indicate that the improvement effect of CL and PG is dosage dependent. At low dosages (e.g., 0.5–2.5% and 4% for CL and PG, respectively), the soil maintains its dispersive nature or shows only slight improvement. This is primarily because the amounts of CL and PG added were insufficient to substantial alter the inherent properties of the soil, resulting in some soil particles still being able to disperse in water. However, at higher dosages (e.g., 4.5–8% and above), the soil gradually transforms into nondispersive soil. This is mainly due to the sufficient addition of Ca^2+^ from CL into the dispersive soil, inducing phase separation and the formation of calcium aluminate silicate hydrate (C–S–H) and geopolymer gels (C–A–S–H) [20]. The OH^−^ in CL provides an alkaline condition that promotes the dissolution of silicates and aluminates from volcanic ash materials, forming gels with adhesive properties.

#### 3.1.2. XRD Test

**Figure 5:**
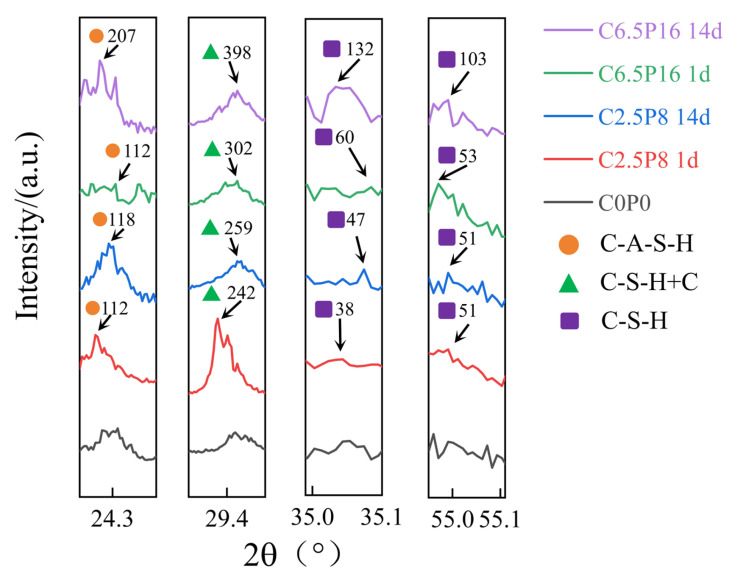
XRD spectra of stabilized soils with different dosages and curing ages. Peaks are indexed with reference to the following ICDD PDF cards: quartz (Q): 00-046-1045; calcite (C): 01-086-2334; montmorillonite (M): 00-058-2028; illite (I): 00-026-0911; kaolinite (K): 00-001-0527; albite (A): 00-009-0465; potassium feldspar (F): 00-025-0618.

Figure 5 illustrates the XRD patterns of PG/CL compacted mixtures at two curing ages (1 day and 14 days). As observed from the figure, the main mineral components of the dispersive soil samples consist of clay minerals such as montmorillonite (M) and illite (I) and nonclay minerals such as kaolinite (K), albite (A), orthoclase (F), calcite (C) and quartz (Q) [21]. The dispersibility of dispersive soils is typically closely related to their mineral composition and the total dissolved salt content [22,23]. High concentrations of Na^+^ ions destabilize the structure of dispersive soils, increasing the risk of dispersion and cracking within the soil [24]. Changes in peak values and the emergence of new peaks were observed in the XRD spectra when 2.5% CL and 8% PG were added to the dispersive soil. This indicates that complex ion exchange reactions and pozzolanic reactions occurred, with low-valent Na^+^ and K^+^ ions being replaced by Ca^2+^ and Al^3+^, thereby reducing the thickness of the double electrical layer of soil particles and enhancing the aggregation force between particles. At the same time, cementitious products such as C–S–H and geopolymers such as C–A–S–H were formed. The concentration of soluble ions (e.g., OH^−^ and Ca^2+^) in the samples increased as the content of CL and PG increased. Under alkaline conditions, silica and alumina are more easily dissolved and calcium ions react with these dissolved elements to form C–S–H and C–A–S–H [25]. This suggests that the formation of cementitious products facilitated the flocculation and cementation between soil particles, thereby reducing soil dispersibility. Furthermore, as illustrated in this figure, a new calcite phase was detected in the C6.5P16 sample after 14 days of curing (2θ = 47.5°, θ: the angle between the incident X-ray and the lattice plane.). This may be attributed to the incomplete involvement of 6.5% CL in the pozzolanic reaction or ion exchange, which instead reacted with carbon dioxide in the air, resulting in the formation of calcium carbonate through carbonation [26]. Although carbonation may have occurred, it is also possible that the calcite originated from residuals in the CL.

#### 3.1.3. Effect of Curing Age

**Figure 6:**
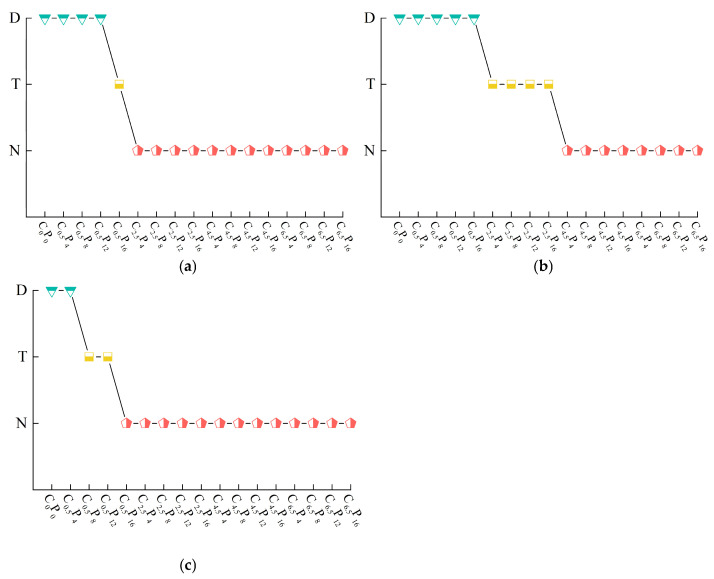
Comprehensive classification results of dispersibility of stabilized soil: (**a**) curing age of 1 day; (**b**) curing age of 7 days; (**c**) curing age of 14 days. **Note:** D, T, and N represent dispersive soil, transitional soil, and nondispersive soil, respectively.

As illustrated in Figure 6, the comprehensive determination results of the dispersibility test indicate that calcium-based composite materials have a positive impact on the dispersibility of the samples to a certain extent. For instance, increasing the curing period does not reduce the dispersibility of the samples when the amounts of CL and PG in the calcium-based composite materials are 0.5% and 4%, respectively. This suggests that the curing effect is not substantial at lower dosages of calcium-based composite materials. The dispersibility of the samples is effectively improved with the extension of curing period when the amounts of CL and PG are 0.5% and 4–12%, respectively. However, the trend is not very pronounced. The dispersibility of the samples shows an overall decreasing trend with the prolongation of curing age when the amounts of CL and PG are 0.5% and 16%, respectively. Furthermore, the dispersibility of the samples is completely eliminated at CL and PG dosages of 2.5% and 4–16%, respectively; however, a certain reaction time is required. The improvement in curing effect becomes more apparent when the amounts of CL and PG are 4.5–6.5% and 4–16%, respectively and the samples exhibit nondispersive characteristics at all curing periods.

It should be noted that while higher PG content (up to 16%) showed benefits in this study, excessive PG might lead to reduced workability, increased cost, or insufficient alkaline activation if the CL content is not proportionally increased. The optimal range identified (CL 4.5–6.5%, PG 4–16%) balances effectiveness with practical applicability.

It can be observed that with the increase in curing period, calcium hydroxide (Ca(OH)_2_) gradually transforms into more stable hydration products such as calcium silicate hydrate C–S–H and C–A–S–H. Therefore, prolonged curing period facilitates the occurrence of more chemical reactions, resulting in the formation of additional hydration products that further enhance the soil improvement effect [20]. As curing period increases, the C–S–H and C–A–S–H gels generated from the reactions between PG and quicklime gradually form a denser network structure [21]. This structure tightly bonds the soil particles, improving the stability and strength of the soil. Within a shorter curing period, the reaction between calcium hydroxide and PG proceeds rapidly but is not complete. To achieve better improvement effects, a longer curing period is required for the reactions to fully take place. Thus, extending the curing period enables the completion of more reactions, thereby enhancing the improvement effect.

#### 3.1.4. Permeability Test

**Figure 7:**
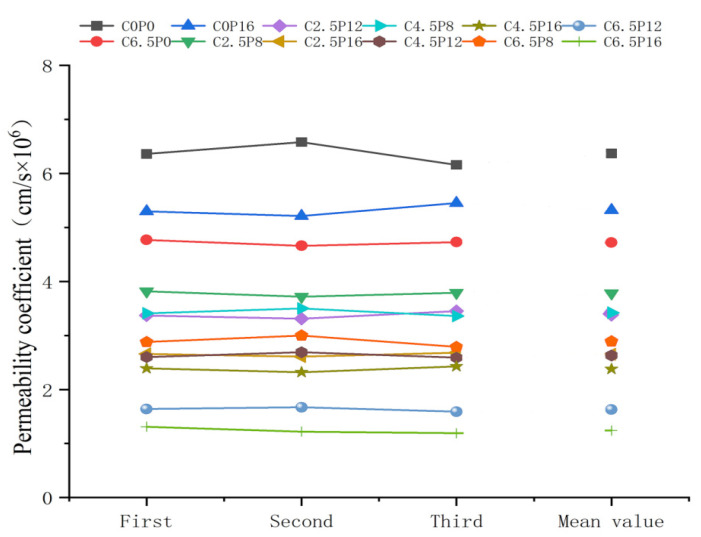
Relationship between the amount of curing soil and the permeability coefficient.

The results of the permeability coefficients for different samples are depicted in Figure 7. As illustrated in the figure, the permeability coefficient generally exhibits a decreasing trend as the amounts of CL and PG increase. The permeability coefficient of C0P0 is 6.37 × 10^−6^ cm/s, while that of C6.5P0 decreases to 4.72 × 10^−6^ cm/s and that of C0P16 drops to 5.32 × 10^−6^ cm/s. When the PG content is 16%, increasing the amount of CL from 2.5% to 6.5% reduces its permeability coefficient from 2.65 × 10^−6^ cm/s to 1.24 × 10^−6^ cm/s. This indicates that the increase in the amount of CL has a substantial effect on decreasing the permeability coefficient. Furthermore, the amount of PG also has a notable impact on the permeability coefficient. When the amount of CL remains constant, an increase in the amount of PG leads to a marked decrease in the permeability coefficient. For instance, during the transition from C2.5P8 to C2.5P16, the permeability coefficient decreases from 3.78 × 10^−6^ cm/s to 2.65 × 10^−6^ cm/s, demonstrating that the increase in the amount of PG leads to a reduction in permeability coefficient.

The observed experimental phenomena can be attributed to the fact that with the increase in the addition of modifier, the hydration products (such as C–S–H and C–A–S–H gels) formed from the reaction between PG and CL play a key role. These products not only fill the voids between soil particles, improving soil compaction, but also enhance the cohesion between particles, reducing the water flow pathways within the pores. As the amount of materials increases, the quantity of these hydration products also increases, further suppressing the flow of moisture and lowering the permeability coefficient of the soil [20]. Therefore, a rational dosage adjustment of the calcium-based composite materials can optimize the permeability performance of the soil. The permeability coefficient of the cured soil substantially decreases at higher dosages of CL and PG, indicating that the calcium-based composite material has a favorable effect on improving the permeability characteristics of the soil.

### 3.2. Microscopy and Phase Composition

#### 3.2.1. SEM Test

**Figure 8:**
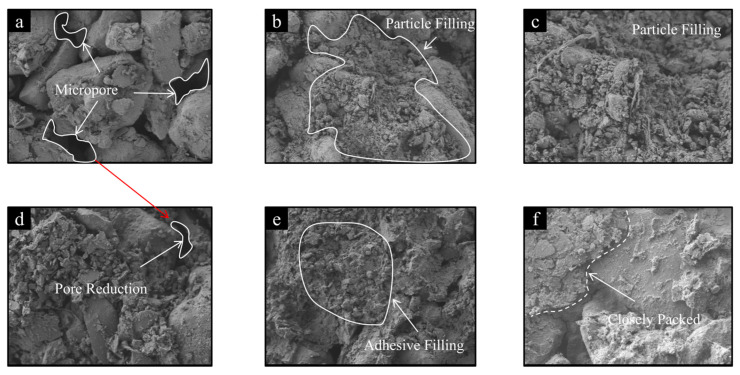
Microstructure of stabilized soil under different binder contents and curing ages: (**a**) C0P0 at 500× magnifications; (**b**) C2.5P8 cured for 1 day at 500× magnifications; (**c**) C2.5P8 cured for 1 day at 1000× magnifications; (**d**) C6.5P16 cured for 1 day at 1000× magnifications; (**e**) C2.5P8 cured for 14 days at 500× magnifications; (**f**) C6.5P16 cured for 14 days at 1000× magnifications.

Figure 8 shows the SEM images of soil samples with different binder contents and curing ages. The untreated dispersive soil sample, without the addition of calcium-based composite materials, exhibits a loose, mesh-like structure with large, well-connected pores and minimal particle-to-particle contact, resulting in an overall loose configuration (Figure 8a). It can be observed that there is a lack of effective cementing agents between the soil particles; contacts are primarily point-to-point or point-to-face and the pores remain largely unfilled.

With the increasing dosage of lime and glass powder, significant changes occur in the soil’s microstructure. At low dosages (e.g., 2.5% lime and 8% glass powder), a small amount of cementitious material begins to form. The inter-particle pores are gradually filled and some fine particles adhere to the surfaces of larger particles, contributing to partial pore filling (Figure 8b).

When the lime dosage reaches 4.5% or more and the glass powder dosage reaches 4% or more, the soil microstructure becomes considerably denser. The contact mode between particles shifts from edge-to-edge to face-to-face, with particle boundaries becoming blurred, indicating stronger inter-particle bonding (Figure 8e). This is attributed to the increased release of Ca^2+^ ions with higher dosages of calcium-based composites, leading to ion exchange, a reduction in inter-particle repulsive forces and gradual pore densification. The cementitious materials generated by pozzolanic reactions enhance the cohesion between soil particles, thereby improving the soil’s mechanical properties. Some insufficiently reacted fine particles coagulate and adhere to larger particles, causing the dispersed soil particles to aggregate and form new assemblages. This process significantly reduces the number of pores, consequently enhancing the soil’s deformation resistance and bearing capacity.

Based on the SEM analysis, the incorporation of calcium-based composites and the extension of the curing period markedly alter the microstructure of the dispersive soil. The added stabilizing materials generate cementing products through ion exchange and hydration reactions, which fill the pores between soil particles and strengthen the inter-particle bonds. Simultaneously, the chemical reactions increase the strength of attractive forces, leading to a reduced thickness of the diffuse double layer and a lower dispersion potential of soil particles. This helps prevent the particles from being washed away by flowing water and decreases the soil’s erosion rate. The prolonged curing period further promotes these reactions, resulting in a denser and more stable microstructure. These microstructural changes not only reduce the soil’s dispersivity but are also expected to contribute to improved mechanical performance (e.g., strength and erosion resistance), as strongly suggested by the significant reduction in permeability (Section 3.1.4). This microstructural evolution provides a scientific basis for understanding the improved engineering behavior of the stabilized soils.

#### 3.2.2. Correlation Between Permeability and Cementitious Products

**Figure 9:**
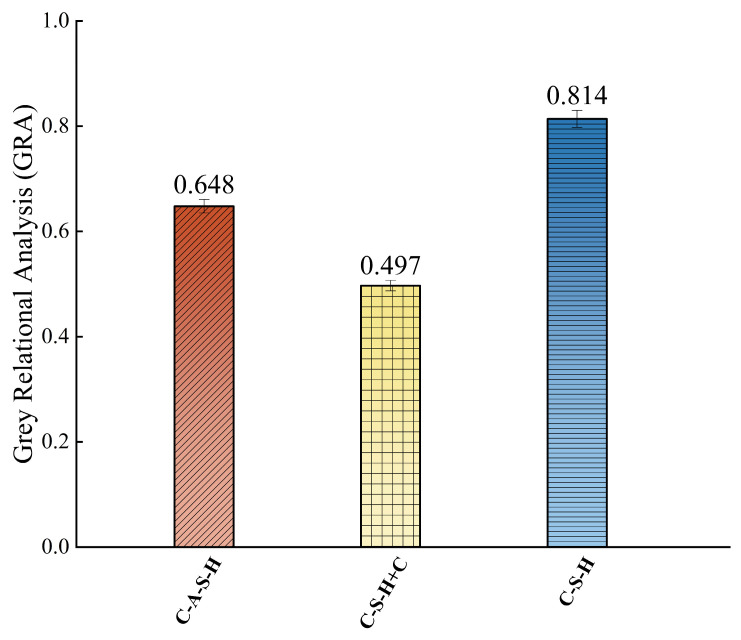
Correlation between permeability and the cementitious products.

According to the calculation of Equations (3)–(7), the final result is shown in Figure 9. There are distinct differences in the relational degrees between the three cement hydration products and the permeability coefficient, reflecting their varying levels of influence on the material’s permeability. Among them, C-S-H exhibits the highest relational degree with the permeability coefficient, reaching 0.814, indicating that it has the most significant impact on permeability among all the analyzed components and may serve as a key product determining permeability performance. C-A-S-H shows a relational degree of 0.648, suggesting a moderate influence, though relatively weaker. In contrast, C-S-H+C has the lowest relational degree of only 0.497, implying that this component has a relatively minor direct effect on the permeability coefficient, or its role in the system may be regulated by other factors.

#### 3.2.3. Pore Directionality

For a quantitative analysis of the soil structure, the microstructure of the stabilized soil in SEM images was evaluated using the PCAS software (version 2.322), following a three-step procedure: (1) adjusting the grayscale threshold to enhance the contrast between particles and pores; (2) performing binarization, during which the software automatically removes scattered noise and identifies particles and pores; and (3) outputting geometric and statistical parameters of the pores (or particles), including their number, area, perimeter, length, width and orientation. Through this approach, a geometrically visualized representation of the loess pore structure was achieved based on the PCAS software.

**Figure 10:**
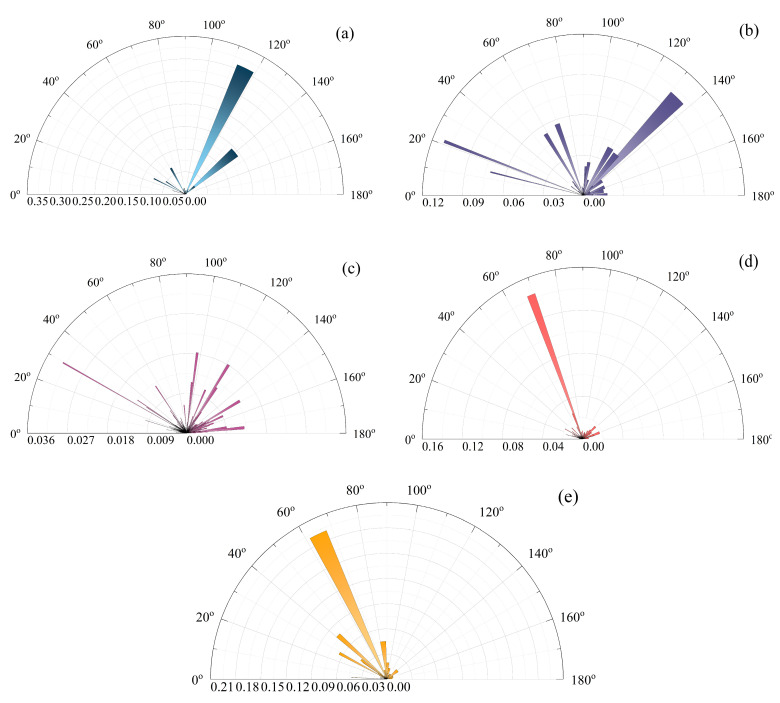
Pore orientation in different soil samples: (**a**) C0P0; (**b**) C2.5P8 cured for 1 day; (**c**) C6.5P16 cured for 1 day; (**d**) C2.5P8 cured for 14 days; (**e**) C6.5P16 cured for 14 days.

Figure 10 illustrates the variation in pore orientation of the stabilized soil under different initial conditions. The pore rose diagrams clearly indicate that the directional distribution of pores becomes more pronounced with increasing binder content or curing age. For instance, in the sample with a mix proportion of C6.5P16 cured for 1 day, the pore orientation ranges between 27° and 30° and 96°–98°. When the curing age reaches 14 days, the pore orientation becomes relatively concentrated within 61°–68°, indicating a more aligned pore structure. This phenomenon can be attributed to the binding and filling effects of the cementitious products formed by the calcium-based composite materials, which occupy interparticle pores and partially eliminate them. Meanwhile, ion exchange reduces interparticle repulsion, promoting a more stable particle arrangement. As a result, most of the remaining pores tend to align along certain preferred orientations.

**Figure 11:**
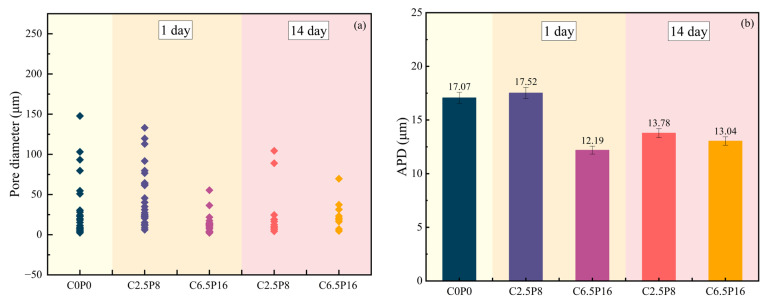
Variation in pore size distribution of stabilized soil: (**a**) C0P0 C2.5P8 and C6.5P16 cured for 1 and 14 days; (**b**) average pore diameter.

Figure 11 illustrates the variation in pore size distribution of stabilized dispersive soil samples under different binder contents and curing ages. It can be clearly observed from the figure that the binder content has a significant influence on the pore size distribution of the soil samples. Taking the samples cured for 1 day as an example, with increasing binder content, the pore size distribution range gradually narrows from 0 to 133.12 μm to 0–55.39 μm, while the corresponding average pore diameter (APD) decreases from 17.52 μm to 12.19 μm, indicating that higher binder content suppresses the formation of large pores.

Meanwhile, as evidenced by the previously conducted SEM imaging, the extension of the curing age also exerts an important effect on the evolution of the pore structure, manifested by a notable reduction in the proportion of large-scale pores. For instance, in the soil sample with a mix proportion of C2.5P8, as the curing age increases, the pore size distribution shifts from 0 to 133.12 μm to 0–104.37 μm, accompanied by a reduction in APD of 3.74 μm, suggesting that continued hydration leads to progressive refinement of the pore structure.

The observed trends in pore size distribution are consistent with those reflected in the pore area distribution, further confirming that the evolution of pore diameter in stabilized soil is an important factor influencing the development of its mechanical properties. In addition, statistical analysis reveals a significant linear negative correlation between the APD of all pores in the specimens and either the binder content or the curing age. This indicates that increased binder content or prolonged curing promotes the transformation of large-scale pores into smaller ones, thereby enhancing the compactness of the soil structure. These results provide direct visual evidence of the microstructural densification of the modified soil, thereby corroborating the theory that the pores are filled by C-S-H and C-A-S-H gels.

### 3.3. Enhancement Mechanism of the Dispersive Soils

**Figure 12:**
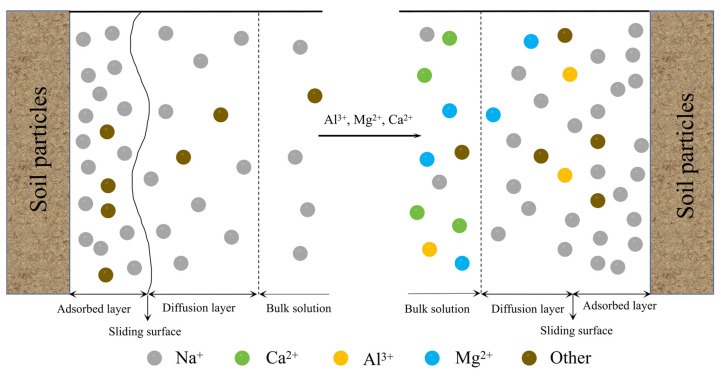
Schematic diagram of the ion exchange process.

The modification process and mechanism of calcium-based composite materials for stabilizing dispersive soil are analyzed and discussed based on the characteristics of the dispersive soil in the Lei He Zui Reservoir area and the composite effect of CL and PG. Figure 12 illustrates the schematic diagram of the ion exchange process. The dispersive soil contains a relatively high concentration of Na^+^ and the hydration layer of Na^+^ is comparatively thick. The thickness of the electric double layer around Na+ containing soil particles is substantially greater than that of the double layers formed by other common cations (such as Ca^2+^, Mg^2+^ and Al^3+^) [27]. Such soil particles exhibit a high degree of dispersivity. Calcium-based composite materials can be incorporated to release a substantial amount of soluble Ca^2+^, Mg^2+^ and Al^3+^ into the pore solution of the soil. According to cation exchange theory, the Na^+^ ions adsorbed on the surface of soil particles can be replaced by these soluble cations [22]. According to the charge balance theory, the thickness of the electric double layer will rapidly decrease after Na^+^ ions on the surface of soil particles are replaced by substantial amounts of soluble Ca^2+^, Mg^2+^ and Al^3+^. This results in a substantial reduction in the thickness of the water film surrounding the soil particles, thereby decreasing the distance and gap between soil particles [28,29].

The soil–water reaction process after CL treatment is illustrated in Equations (8) and (9). This phenomenon indicates that CL and PG can improve the stability of dispersive soils by promoting the flocculation or aggregation of soil particles, thereby altering the soil structure. CL serves as an effective stabilizer, offering an alternative to lime [30].

(8) $$nCa{\left(OH\right)}_{2}+nAl_{2}O_{3}\to nCaO\cdot Al_{2}O_{3}+nH_{2}O$$

(9) $$nCa{\left(OH\right)}_{2}+nSiO_{2}+nH_{2}O\to nCaO\cdot SiO_{2}+2nH_{2}O$$

**Figure 13:**
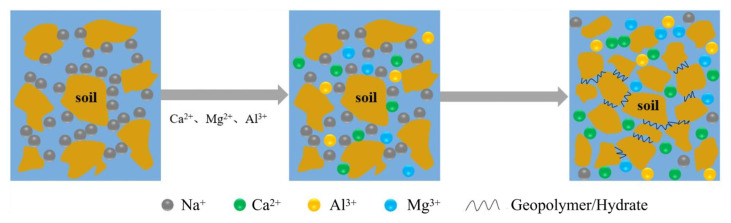
The modification process and mechanism of dispersive soil by calcium-based composite materials.

Figure 13 illustrates the modification mechanism of dispersive soil by calcium-based composite materials. With the extension of curing period, the amount of C–S–H and C–A–S–H geopolymer gels gradually increases. The corresponding chemical reactions are depicted in Equations (10) and (11). These cementing materials gradually bind the adjacent soil particles together, forming a connected structure that disrupts the percolation pathways of free soil particles and restricts their free movement. Furthermore, the products of the ion exchange reactions work synergistically with the hydration products and geopolymers, enhancing the water stability of the soil sample and reducing its dispersivity [31]. Therefore, a specific curing period is required as a condition to enhance the structural strength of the soil and inhibit its dispersivity.

(10) $$Ca^{2+}+SiO_{2}+OH^{-}+(n-1)H_{2}O\to CaO\cdot SiO_{2}\cdot nH_{2}O$$

(11) $${\left(Al,Si\right)}_{2}Compand+H_{2}O+Ca{\left(OH\right)}_{2}\to Ca{\left[-{\left(SiO_{2}\right)}_{y}-AlO_{2}-\right]}_{n}\cdot mH_{2}O$$

## 4. Summary and Conclusions

The novelty of this study lies in the systematic investigation of the binary CL-PG composite system for dispersive soil stabilization, which remains scarcely reported. It quantitatively clarifies the synergistic effect, optimal mix ratio, and the correlative mechanisms between microstructural evolution and macroscopic performance (dispersibility and permeability), filling a gap in the existing literature. This study not only provides a novel technical pathway for the high-value utilization of CL and PG but also offers important theoretical and experimental foundations for developing high-performance, low-environmental-impact green composites in geotechnical engineering. The main findings are:

(1) Scientific Contribution and Novelty: The CL-PG composite demonstrates a distinct synergistic effect. The binary system is shown to be more effective in suppressing dispersibility and reducing permeability than the use of single components, due to the coupled ion exchange and enhanced pozzolanic reactions.
(2) Optimal Dosage for Dispersibility Control: A clear threshold exists: CL ≥ 2.5% and PG ≥ 8% are required for significant improvement. The composite with CL % and PG between 4–16% renders the soil non-dispersive across all tested curing ages (1–14 days), providing a critical optimal mix ratio for engineering applications.
(3) Permeability Reduction Mechanism: The permeability coefficient decreases markedly with increasing CL and PG content. This is directly correlated with microstructural densification, as evidenced by SEM and pore analysis, resulting from the formation of C-S-H and C-A-S-H gels.
(4) Underlying Mechanisms: The stabilization mechanism is twofold: (i) Ca^2+^ from CL promotes ion exchange, reducing the double-layer thickness and flocculating soil particles; (ii) The alkaline environment activates SiO_2_ and Al_2_O_3_ from PG, leading to pozzolanic reactions that generate cementitious gels (C-S-H, C-A-S-H), which fill pores and enhance interparticle bonding.

This work confirms the high potential of the CL-PG composite as a sustainable and effective soil stabilizer, transforming two waste streams into valuable geotechnical materials.

## Figures and Tables

**Table 1:** Basic physical properties of soil samples.

Sample	$G_{S}$ (g/cm^3^)	$w_{L}$ (%)	$w_{P}$ (%)	$I_{p}$ (%)	Particle Composition (%)			$\rho_{d}$ (g/cm^3^)	OMC (%)	Soil Classification
					Sand (mm)	Silt (mm)	Clay (mm)			
Test soil	2.68	26.4	15.0	11.4	27.81	65.72	6.47	1.71	14.2	Low liquid-limit clay

**Note:** $G_{S}$, particle relative density; $w_{L}$, liquid limit; $w_{P}$, plastic limit; $I_{p}$, plasticity index; $\rho_{d}$, maximum dry density; OMC, optimum moisture content.

**Table 2:** Mineral composition of dispersive soil samples in the study area.

Mineral Composition	I/S	I	K	Total Clay Mineral Content (%)	Q	C	P
Content (%)	3.6	18.2	2.8	24.9	43.9	9.6	18.1

**Note: I/S**, illite–smectite mixed layer; **I**, illite; K, kaolinite; **Q**, quartz; **C**, calcite; **P**, plagioclase.

**Table 3:** Sample dispersibility classification test plan.

Test Category	CL (%)	PG (%)	Curing Age (days)	Compaction Degree (%)
Pinhole test	0.5, 2.5, 4.5, 6.5	4, 8, 12, 16	1, 7, 14	95
Mud ball test				
Permeability test				
Diffraction test	2.5, 6.5	8, 16	1, 14	98

## Data Availability

The original contributions presented in this study are included in the article. Further inquiries can be directed to the corresponding author.

## Abbreviations

$G_{S}$	Particle relative density
$w_{L}$	Liquid limit
$w_{P}$	Plastic limit
$I_{p}$	Plasticity index
$\rho_{d}$	Maximum dry density
OMC	Optimum moisture content.
I/S	Illite–smectite mixed layer
I	Illite
K	Kaolinite
Q	Quartz
C	Calcite
P	Plagioclase
XRD	X-ray diffraction
D	Dispersive soil
T	Transitional soil
N	Non-dispersive soil
SEM	Scanning electron microscope
A	Albite
F	Potassium feldspar
C–S–H	Calcium silicate hydrate
C–A–S–H	Calcium aluminate silicate hydrate
